# Using AI-Based Virtual Simulated Patients for Training in Psychopathological Interviewing: Cross-Sectional Observational Study

**DOI:** 10.2196/78857

**Published:** 2025-12-23

**Authors:** Daniel García-Torres, César Fernández, José Joaquín Mira, Alexandra Morales, María Asunción Vicente

**Affiliations:** 1 Departamento de Psicología de la Salud Universidad Miguel Hernández Elche Spain; 2 Fundación para el Fomento de la Investigación Sanitaria y Biomédica de la Comunitat Valenciana Alicante Spain

**Keywords:** artificial intelligence, clinical reasoning, educational technology, natural language processing, patient simulation, psychopathology, simulation training

## Abstract

**Background:**

Virtual simulated patients (VSPs) powered by generative artificial intelligence (GAI) offer a promising tool for training clinical interviewing skills; yet, little is known about how different system- and user-level variables shape students’ perceptions of these interactions.

**Objective:**

We aim to study psychology students’ perceptions of GAI-driven VSPs and examine how demographic factors, system parameters, and interaction characteristics influence such perceptions.

**Methods:**

We conducted a total of 1832 recorded interactions involving 156 psychology students with 13 GAI-generated VSPs configured with varying temperature settings (0.1, 0.5, 0.9). For each student, we collected age and sex; for each interview, we recorded interview length (total number of question–answer turns), number of connectivity failures, the specific VSP consulted, and the model temperature. After every interview, students provided a 1-10 global rating and open-ended comments regarding strengths and areas for improvement. At the end of the training sequence, they also reported perceived improvement in diagnostic ability. Statistical analyses assessed the influence of different variables on global ratings: demographics, interaction-level data, and GAI temperature setting. Sentiment analysis was conducted to evaluate the VSPs’ clinical realism.

**Results:**

Statistical analysis showed that female students rated the tool significantly higher (mean rating 9.25/10) than male students (mean rating 8.94/10; Kruskal-Wallis test, H=8.7; *P*=.003). On the other side, no significant correlation was found between global rating and age (*r*=0.02, 95% CI –0.03 to 0.06; *P*=.42), interview length (*r*=0.04, 95% CI –0.2 to 0.10; *P*=.18), or frequency of participation (Kruskal-Wallis test, H=4.62; *P*=.20). A moderate negative correlation emerged between connectivity failures and ratings (*r*=–0.26, 95% CI –0.41 to –0.10; *P*=.002). Temperature settings significantly influenced ratings (Kruskal-Wallis test, H=6.93; *P*=.03; η²=0.02), with higher scores at temperature 0.9 compared with 0.1 (Dunn’s test, *P*=.04). Concerning learning outcomes, self-perceived improvement in diagnostic ability was reported by 94% (94/100) of students; however, final practical examination scores (mean 6.67, SD 1.42) did not differ significantly from those of the previous cohort without VSP training (mean 6.42, SD 1.56). Sentiment analysis indicated predominantly negative sentiment in GAI responses (median negativity 0.8903, IQR 0.306-0.961), consistent with clinical realism.

**Conclusions:**

GAI-driven VSPs were well-received by psychology students, with student gender and system-level variables (particularly temperature settings and connection stability) shaping user evaluations. Although participants perceived the training as beneficial for their diagnostic skills, objective examination performance did not significantly differ from the previous cohort. However, lack of randomization limits the generalization of the results obtained, and further experiments are required.

## Introduction

In health education, the development of clinical reasoning is fundamental for preparing competent professionals capable of making accurate diagnostic and therapeutic decisions. However, formal instruction in clinical reasoning remains limited within many curricula, often due to time constraints and the lack of targeted pedagogical approaches. As a result, recent graduates frequently report feeling inadequately prepared to manage the ambiguity and complexity inherent in real-world clinical practice, particularly in clinical psychology, where effective diagnostic formulation requires integrating diverse, nuanced patient information [1,2].

Clinical skill development in psychology education, particularly in subjects such as psychopathology, presents a significant challenge for university programs. Successful clinical training necessitates the integration of theoretical knowledge—such as diagnostic criteria—and practical skills, such as conducting clinical interviews. Acquiring competencies such as symptom identification, differential diagnosis, clinical reasoning, and empathic communication extends beyond theoretical understanding. These competencies are deeply intertwined with practical experience, decision-making in uncertain contexts, and sustained exposure to complex clinical situations. Unfortunately, traditional teaching methods, such as paper-based clinical cases, offer limited opportunities for students to actively and progressively develop these skills, negatively affecting their confidence and preparedness.

To address these limitations, the use of virtual patients has increasingly emerged as an effective pedagogical strategy [3,4], offering simulations of realistic clinical encounters in a risk-free environment. These simulations allow students to practice crucial skills such as history taking, hypothesis formulation, and diagnostic reasoning without risking patient safety [5,6]. Virtual patient technologies have evolved considerably—from initial static textual cases to sophisticated interactive simulations powered by generative artificial intelligence (GAI) and natural language processing (NLP) technologies [3].

The integration of GAIs based on large language models (LLMs), such as ChatGPT into virtual patient platforms represents a significant advancement in educational simulations. These models facilitate realistic, responsive interactions that closely resemble genuine clinical dialogues, thereby increasing learner engagement and immersion [7]. Recent studies, including a systematic review, have shown that GAI-powered conversational virtual patients (virtual simulated patients [VSPs]) significantly enhance clinical reasoning skills and student satisfaction, especially when the interactions are perceived as authentic and dynamic [8].

Concerning authenticity, LLMs are parameterizable in different ways to adjust their behavior. In particular, the temperature parameter controls how random or deterministic LLMs’ choices are: low temperature values produce more predictable and less spontaneous answers, whereas high temperature values produce more creative and natural-sounding answers (although less consistent). This effect is discussed in detail in the report presented by Peeperkorn et al [9]. Temperature control is thus relevant in a VSP, where natural-sounding answers are preferable, but consistency is also a requirement.

Despite the promising literature on VSPs, existing research has predominantly focused on medical education (eg, Peralta Ramírez et al [10] or Borg et al [11]) and nursing education (eg, Padilha et al [12] or Hu et al [13]). There remains a gap regarding their effectiveness in psychology education, particularly in the field of psychopathology. A complete review of VSP applications in psychology can be found in Imam Hossain et al [14]. Among the few previous studies in this field, the work by Lan et al [15] proposes an alternative to objective structured clinical examinations in psychology based on VSPs, which, however, are not powered by GAI. Another study from Walkiewicz et al [16] compares actors or standardized patients with VSPs, the main conclusion being that standardized patients were more effective for interview skills and VSPs were most effective for clinical reasoning skills. Also in this case, the VSP platform used was not powered by a GAI.

This study evaluates the students’ perceptions of GAI-based VSPs for practical psychopathology training in an undergraduate psychology course of a public Spanish University.

## Methods

### Experimental Design

This study used a cross-sectional observational design to evaluate the effectiveness of GAI-based VSPs in training psychological diagnostic skills.

Every student-VSP session followed a similar schedule: the student started with no prior knowledge about the case, except from the name and age of the patient (eg, a session may start with a heading like “Simon, a 12-year-old boy, is your new patient”). With only this limited information, the student had to start the interview with the patient and ask all questions she or he found necessary to reach a conclusion about a diagnosis for the patient. When the student had gathered all information needed, she or he ended the interview and filled out a report specifying the diagnose and, depending on the patient, answering a set of extra questions related to the case.

Apart from that, the student also rated the tool after each session and evaluated self-perceived learning improvement. All sessions ended through 2 web-based questionnaires. Both questionnaires adhere to the Checklist for Reporting Results of Internet E-Surveys (CHERRIES) guidelines [17] (Multimedia Appendix 1).

The first questionnaire (student satisfaction, completed after each interview) consisted of 3 items, distributed across a single screen (page). The second questionnaire (learning improvement, completed only once after all practice sessions) had 14 questions distributed across 5 screens (pages), although only one of these items is included in this study. The project team was multidisciplinary: the psychologists designed both questionnaires, and the engineers designed the responsive web application following this design and assuring correct behavior on different screen sizes.

The study was conducted as a “closed survey,” requiring participants to log in via the university’s virtual campus with their unique student credentials. Once the questionnaire had been submitted, the students could check their answers and the conversation with the VSP, but the submit button was disabled to prevent duplicate entries. Furthermore, the application only allowed the submission of fully completed questionnaires. To remove nonmeaningful interactions from the dataset, sessions with fewer than 3 questions in the conversation between student and VSP were excluded from analysis.

In selected sessions, the GAI model’s temperature parameter was fixed randomly at one of 3 different levels: 0.1, 0.5, and 0.9. This setting was unknown to the students in all cases. As outlined in the Introduction section, temperature controls the degree of randomness in the model’s responses: lower values (eg, 0.1) produce more deterministic and structured replies, while higher values (eg, 0.9) allow for more varied and unpredictable outputs. The study explored whether this parameter influenced students’ perceptions of the tool (tool rating), as well as the length of the interviews (number of questions asked by the student).

The platform recorded the complete interaction history, including both student inputs and GAI responses. The length of each interview was measured in terms of the number of questions asked by the student and answered by the VSP. We also explored whether this parameter influenced students’ ratings of the tool.

Due to internet connectivity issues, the GAI model was occasionally unreachable, and certain student questions were not answered by the VSP. In these cases, the message received by the student was “Connection error, please repeat your question.” Interview length did not account such failed interactions. We recorded separately the number of these connectivity failures in every interview to evaluate their possible influence on student ratings.

### Platform Development

The starting point for platform development was 13 cases of different psychopathologies described in terms of (1) symptoms, clinical history, and familial or social context; and (2) questions to be answered by the students, including a proposal of the correct diagnosis for the patient.

The desired final result was 13 GAI-based VSPs behaving accordingly to each of the 13 cases. The VSPs did not offer any initial information about their diseases, and the students were responsible for gathering all information by interviewing them. An important requirement was to allow interaction using unlimited natural language (ie, free text instead of selection from predefined questions). After the interviews, the software had to ask the students the questions related to the case, including the proposal of a correct diagnosis. The complete interview (student questions and VSP answers) had to be registered for further analysis.

Other goals to be fulfilled by the VSP platform included:

- It should enable health care educators without programming expertise to modify and adjust the VSPs.
- The reliability of the GAI responses had to be assured, to avoid hallucinations or incorrect VSP answers to student questions.
- It should allow an easy customization of key GAI parameters—such as temperature (controlling response randomness) and top_p (influencing response diversity).
- It should facilitate user satisfaction assessment by collecting qualitative feedback and improvement suggestions.

The tools selected for VSP development were the PHP programming language and 2 different GAI models (OpenAI and Mistral AI) accessed through their public APIs.

The platform was designed by a multidisciplinary team involving software engineers, psychologists, and docents. We followed a collaborative approach similar to that presented in Fernández et al [18], under an incremental and iterative software development life cycle [19], in which, for each added functionality, we carried out successive steps of development, revision by the complete team, redesign if needed, and validation. This incremental scheme aimed at 6 different development steps:

- Step 1: Working VSP for the first clinical case: must answer all student questions correctly, according to the patient symptoms and expected behavior in terms of expressiveness and feelings.
- Step 2: Working VSP for the first clinical case with adjustable temperature and top_p parameters for answer randomness control.
- Step 3: Working VSP for the first clinical case with closed-loop supervision by a secondary GAI model and temperature or top_p automatic adjustment.
- Step 4: Working VSP for the first clinical case integrated in a teaching and evaluation environment with access control, final questionnaire for students, and practice registration in the database.
- Step 5: Docent tool for creation and edition of VSPs. This tool will further be used to create the 13 required VSPs for each of the 13 cases.
- Step 6: VSPs created for all 13 cases.

A final validation step was carried out, with exhaustive tests performed by the psychologists and docents for each of the 13 VSPs developed, prior to the start of training sessions with the students.

### Recruitment of Participants and Demographic Data Registered

Participants were recruited from second-year undergraduate psychology students enrolled in the psychopathology course at Miguel Hernández University (UMH), Elche, Spain. This mandatory course, part of the second year of the psychology degree program, was delivered during the first semester (October 2024 to January 2025) of the 2024-2025 academic year and carried a workload of 7.5 credits, according to the European Credit Transfer and Accumulation System. All enrolled students were invited to participate in the study, with no exclusion criteria applied. Participation in the study required attendance at least one of the 6 training sessions scheduled, each one involving interaction with 1-3 different VSPs (globally, 13 VSPs distributed across 6 training sessions; more details can be found on the website [20]).

The only demographic data registered for participants were age and gender.

### Student Satisfaction

Upon completion of each session, participants rated their experience on a 1-10 scale. Ratings of exactly 5 were excluded from the analysis, as this value appeared as the default option on the evaluation form. Because it could not be determined whether these responses were selected intentionally or by omission, their inclusion was considered potentially biased. Therefore, they were removed to preserve the validity of the statistical analysis.

Each student was also encouraged to write 2 open-ended comments: the first detailing the positive aspects found in the tool and the second providing improvement suggestions. Multimedia Appendix 2 shows the structure of the questionnaire.

Student satisfaction was analyzed for relationships with frequency of participation (number of interviews carried out by each student), age and gender of the student, length of interviews, VSP interviewed, number of connectivity failures, GAI temperature parameter, and gender pairing. Gender pairing refers to the possible influence on the tool rating of the VSP and the student having the same or different genders. In other words, the goal is to check whether male or female students rated male or female VSPs differently.

### Learning Improvement

Learning improvement was measured both in terms of perceived improvement and in terms of marks obtained by the students, compared to previous years.

For perceived learning improvement, a final questionnaire was completed (optionally) by the students after all VSP sessions had ended. The only item related to learning improvement was: “Do you consider that interacting with virtual patients helped you improve your ability to identify relevant symptoms during the clinical interview?”

The final questionnaire included other items that are out of scope of this study; more details can be found in Morales et al [21]. Multimedia Appendix 3 shows the structure of the questionnaire.

For mark comparison, the marks obtained by the students in courses 2023/2024 and 2024/2025 were compared. Two items were analyzed: the marks obtained by the students in the practice sessions (reflecting how challenging the practices were) and the marks obtained by the students in the final practice examination (reflecting the competencies they acquired). The final practical examination was a paper-based examination in both courses. The training was also similar in both courses, covering the same 13 clinical cases; however, this training was paper-based in course 2023/2024 and VSP-based in course 2024/2025. For the analysis of average session grades, students with zero attendance were excluded, and the mean was calculated using only attended practices, ensuring that absences did not function as “zero” scores and skew the results.

### Sentiment Analysis

A sentiment analysis was performed on both student questions and GAI-generated responses using a Python script [22] and an NLP library, *Pysentimiento* [23]. This analysis classified the emotional tone of the interactions as positive, neutral, or negative, both at the individual exchange level and for the entire conversation.

### Content Analysis

Regarding open-ended comments, an automated content analysis was carried out to extract the most repeated topics from all user comments, both in the set of positive comments (ie, positive aspects found in the tool) and in the set of critical comments (ie, improvement suggestions). The analysis was automated through GAI to extract the most repeated topics and their repetition counts. Similar automations have been tested in Prescott et al [24], with results comparable to those obtained by human coders, particularly in inductive analyses like the one carried out in this study.

### Statistical Details

Excel (version 16.101.3 for MacOS; Microsoft Corp) was used for data storage. Data processing and analysis were conducted using R (version 4.4.2; R Core Team).

Measures of central tendency and dispersion were calculated for quantitative variables, while frequency distributions were computed for categorical variables. Group comparisons were performed using parametric tests when the assumptions of normality were met and nonparametric alternatives when those assumptions could not be satisfied.

To examine the relationship between students’ ratings of the tool and other quantitative variables, Pearson correlation analyses were conducted.

### Ethical Considerations

This study was approved by the Research Ethics Office of UMH (code DPS.CFP.250116). According to the limited personal data registered (only age and gender), the Research Ethics Office considered the study anonymous, that is, it is not possible to identify a participant from these data. Multimedia Appendix 4 shows the ethical approval record.

Results were stored in a password-protected database whose access was restricted to the researchers taking part in the project.

All students accepted an informed consent prior to every VSP session. The conversation with a VSP did not start unless the student read and accepted the terms. The text of the informed consent was made intentionally clear and concise: “The conversation held with the virtual patient, as well as the answers given in the further questionnaire, will be analyzed in aggregated terms, ensuring privacy and anonymity, as part of a research study whose goal is to improve the use of virtual patients for psychology education. Please confirm that you accept the treatment of your conversation and answers under these conditions.”

After all practice sessions ended, a final, global questionnaire was also presented to the students, who were also required to accept a similar informed consent, with the text: “The results obtained in this questionnaire will be analyzed in aggregated terms, ensuring privacy and anonymity, as part of a research study whose goal is to improve the use of virtual patients for psychology education. By sending the questionnaire you accept the treatment of your answers under these conditions.”

Students received no financial compensation for their participation in the study.

## Results

### Platform Developed

According to the incremental and iterative software development life cycle described in the Methods section, different versions of the application were developed, tested, and validated before proceeding to the next development step. Table 1 shows the development process followed, including development and validation dates.

**Table 1:** Incremental development steps for the virtual simulated patient (VSP) platform.

Step	Developed	Validated
Step 1: Working VSP for first case	April 15, 2024	May 9, 2024
Step 2: VSP with temperature and top_p control	May 15, 2024	May 21, 2024
Step 3: VSP with closed loop supervision	June 19, 2024	June 25, 2024
Step 4: VSP integrated in learning environment	July 3, 2024	July 9, 2014
Step 5: Tool for creating and editing VSPs	July 12, 2024	July 24, 2024
Step 6: VSPs created for each of the 13 cases	September 12, 2024	September 25, 2024

The platform was developed as a responsive web application, optimized for seamless use across desktops, tablets, and smartphones, and programmed using PHP [25].

**Figure 1:**
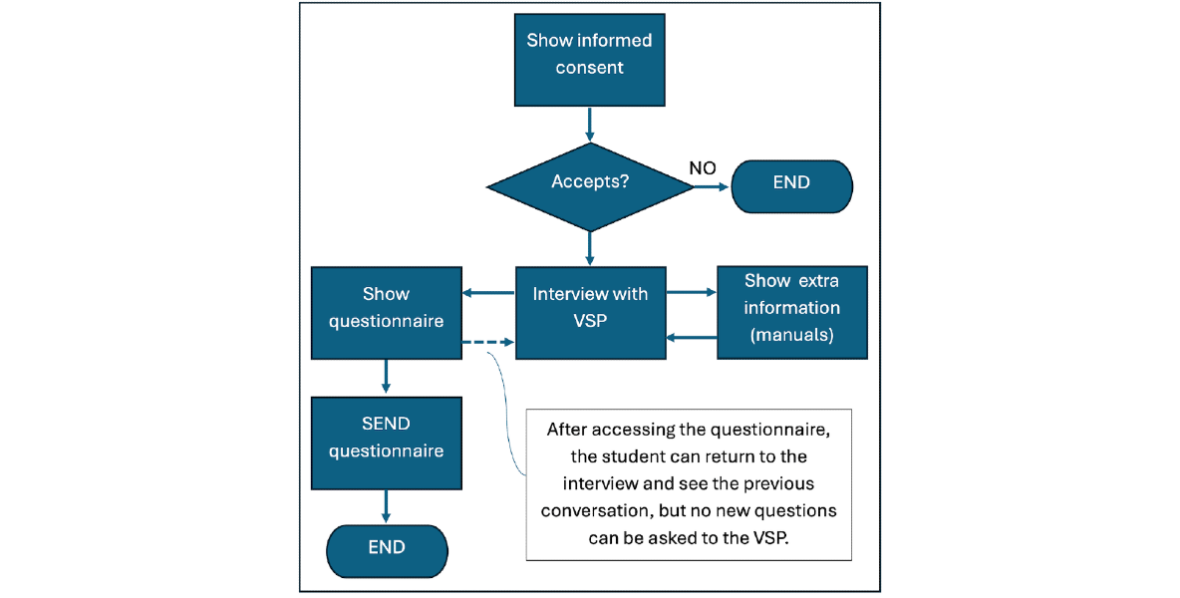
Flowchart of a practice session with a virtual simulated patient (VSP).

Figure 1 shows the flowchart of a practice session, which required initial informed consent. The main screen of the application is the dialogue or interview with the VSP, which can be as complete as the students require (in terms of number of questions asked to the VSP). The students can also check extra information during the practice session, specifically a manual with information on how to diagnose a patient. Once the students access the practice questionnaire, it is allowed to return to the interview screen (to revise the conversation), but it is not allowed to ask new questions to the VSP. After sending the questionnaire with all items fulfilled, the practice session ends.

**Figure 2:**
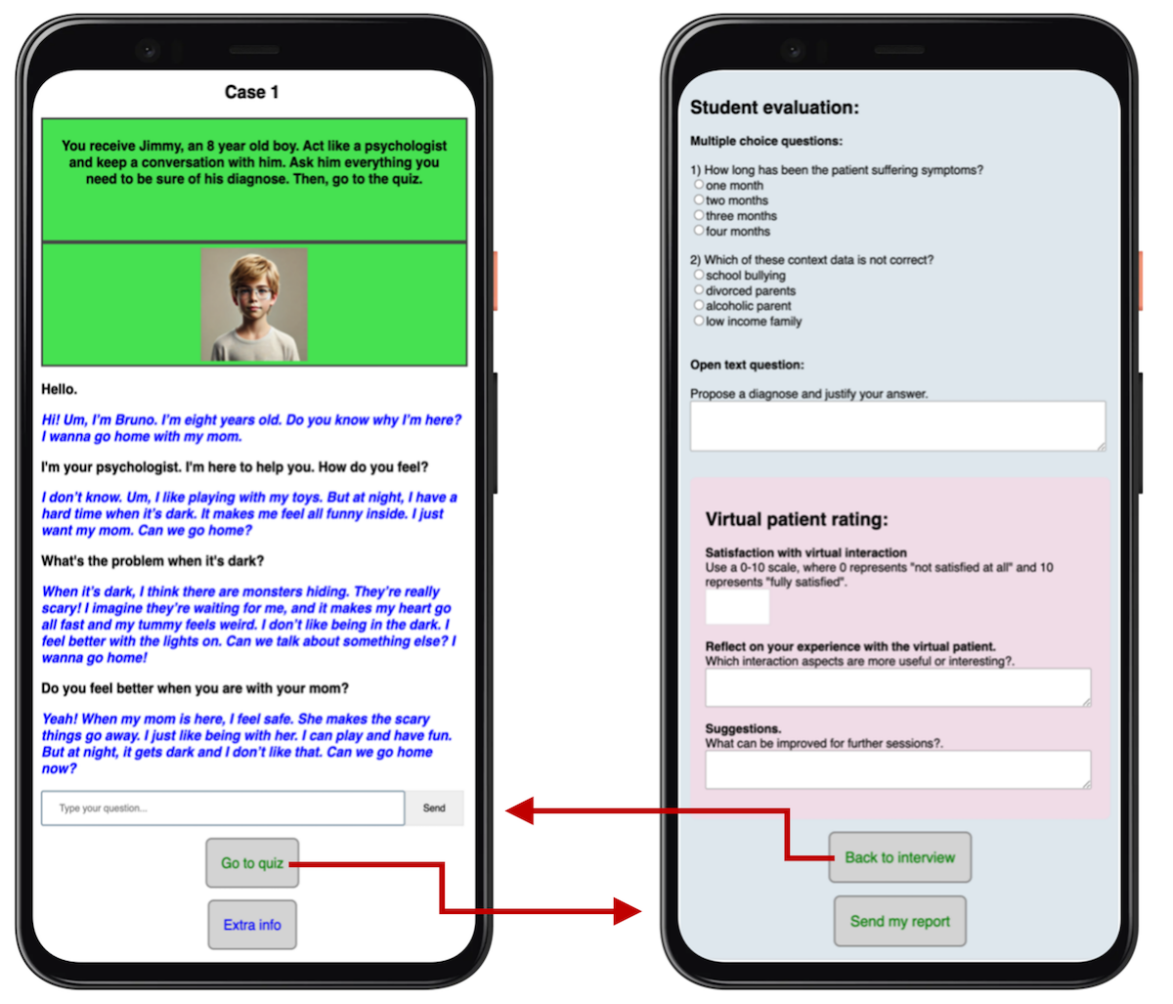
Example screenshots from the virtual simulated patient (VSP) application.

Figure 2 provides example screenshots of a generated VSP interaction: the left-hand image shows the ongoing text-based patient dialogue (ie, interview screen), while the right-hand image presents sample assessment questions provided to the student postinteraction (ie, questionnaire screen).

More details about the VSP platform developed, namely software architecture and the VSP generator for docents, are available in Multimedia Appendix 5.

### Participant Demographics

A total of 156 unique participants took part in the study, carrying out 1832 interviews with VSPs (13 different VSPs). Most of the participants were aged 18-22 years, with a limited number of older participants (3 participants did not provide their ages). Table 2 shows the number of interviews carried out (frequency) per participant age range, among the 153 participants who provided age data.

The sample showed a marked gender imbalance, consisting mostly of female students (127/153, 83%), with male students representing 17% (26/153) of the total sample. Table 2 shows the complete age and gender distribution, which reflects the current trend in Spain, where the number of women enrolled in psychology degree programs significantly exceed that of men, a pattern observed in higher education statistics nationwide (77.2% of female psychology students as of the course 2022/2023 [26], and 79.9% of female psychology graduates [27], preliminary report for the course 2024/2025).

**Table 2:** Frequency and percentage distribution of participants by age range and gender.

Age range (years)	Men, n (%)	Women, n (%)	Total n (%)
18	0 (0)	8 (5.2)	8 (5.2)
19	14 (9.2)	86 (56.2)	100 (65.4)
20	4 (2.6)	10 (6.5)	14 (9.2)
21-25	5 (3.3)	10 (6.5)	15 (9.8)
26-30	1 (0.7)	5 (3.3)	6 (3.9)
31-35	1 (0.7)	4 (2.6)	5 (3.3)
36-40	0 (0)	1 (0.7)	1 (0.7)
41-45	0 (0)	2 (1.3)	2 (1.3)
46-50	1 (0.7)	0 (0)	1 (0.7)
51-55	0 (0)	1 (0.7)	1 (0.7)
Total	26 (17)	127 (83)	153 (100)

### Student Satisfaction

#### Student Satisfaction Versus Demographics and Interview Length

Overall, high ratings (medians close to 10) remained consistent across different demographic groups and interaction levels.

Female students rated the tool significantly higher (mean rating 9.25/10) than male students (mean rating 8.94/10; Kruskal-Wallis test, H=8.7; *P*=.003).

Concerning age, no significant correlation was found between participants’ age and their overall rating of the tool (*r*=0.02, 95% CI –0.03 to 0.06; *P*=.42).

Similar results were obtained for interview length (number of questions posed by participants), with no significant correlation against the overall rating of the tool (*r*=0.04, 95% CI –0.2 to –0.10; *P*=.18). This indicates that the quantity of interaction did not notably influence students’ evaluation of the platform.

**Figure 3:**
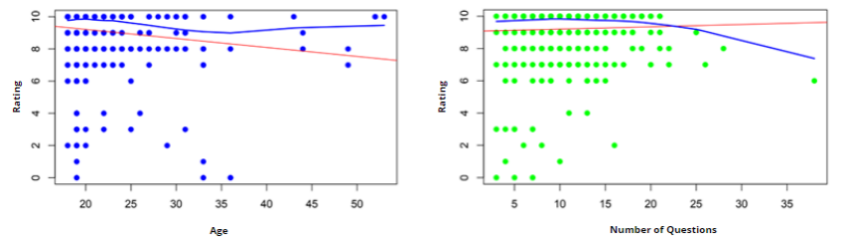
Relationship between participants’ age and their rating of the tool (left panel) and between the number of questions posed and the rating provided (right panel).

Participants age and interview length are plotted against overall ratings in Figure 3. In interpreting these trends, no meaningful association emerged between participants’ age and their rating of the tool: students of different ages consistently evaluated the tool positively, with only minimal variation across the age range. Likewise, although interviews involving a higher number of questions tended to show slightly lower ratings, this pattern was weak and did not indicate a substantial change in students’ perceptions of the tool.

#### Student Satisfaction Versus Frequency of Participation

On average, students rated the tool highly, with minor variations related to their frequency of participation. However, a modest positive trend in average ratings was observed, suggesting that increased exposure might slightly enhance perceptions of the platform’s effectiveness (Table 3). To analyze this relationship between students’ frequency of participation and their average ratings, Shapiro-Wilk tests indicated that ratings did not follow a normal distribution in any of the participation groups (*P*<.001 in all cases). To evaluate whether parametric methods could nevertheless be applied, several common transformations were tested (logarithmic, square root, Box-Cox, and Yeo-Johnson). Although the Yeo-Johnson transformation provided some improvement (eg, W=0.775; *P*<.001 for “Participated once”; W=0.911; *P*=.005 for “6-10 times”), none of the groups achieved normality. Consequently, a nonparametric Kruskal-Wallis test was used as the most appropriate analytic strategy. The results of this test showed that the effect of frequency of participation was not statistically significant (H=4.62, *P*=.20; Table 3).

**Table 3:** Mean ratings of the platform based on student participation frequency (Kruskal-Wallis test, *P*=.20).

Participation frequency	Participants, n	Mean rating (95% CI)
Participated once	16	8.8 (7.3-9.6)
Participated 2-5 times	21	8.9 (8.3-9.4)
Participated 6-10 times	48	9.0 (8.6-9.4)
Participated >10 times	75	9.3 (9.1-9.6)

#### Student Satisfaction Versus VSP Interviewed and Connectivity Failures

**Figure 4:**
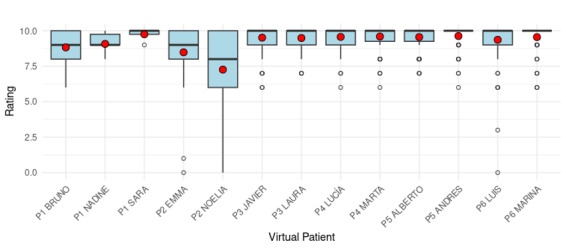
Distribution of participant ratings for each virtual simulated patient (VSP). The red dots represent the mean rating for each VSP. The label “P” indicates the practice session in which each VSP was used (eg, P1=Practice 1).

When analyzing ratings by VSP, overall scores remained high, with most VSPs receiving median values near 10. However, some variation was observed, with median ratings ranging from approximately 8 to 10 across the 13 VSPs (Figure 4). Notably, Emma and Noelia received comparatively lower ratings. These 2 VSPs were involved in a session affected by a higher incidence of internet connectivity issues, which likely contributed to the reduced participant evaluations.

This finding aligns with a moderate negative correlation between the number of internet connectivity issues and participant ratings (*r*=–0.26, 95% CI –0.41 to –0.10; *P*=.002). This suggests that a higher number of connectivity failures was associated with lower ratings from students.

#### Student Satisfaction Versus GAI Temperature Parameter

Shapiro-Wilk tests conducted for each temperature level (0.1, 0.5, and 0.9) indicated strong departures from normality (*P*<.001 in all cases). Attempts to normalize the data through logarithmic and square root transformations were unsuccessful. The Box-Cox procedure suggested a transformation parameter far from 1, while the Yeo-Johnson approach estimated an extreme λ value (λ≈11.2), confirming severe nonnormality. Given these results, nonparametric Kruskal-Wallis tests were again retained as the most suitable analytic approach, revealing a statistically significant difference between them (H=6.93; *P*=.03). The effect size was small (η²=0.02, 95% CI –0.00 to 0.07), suggesting that temperature explained only about 2% of the variance in ratings.

**Figure 5:**
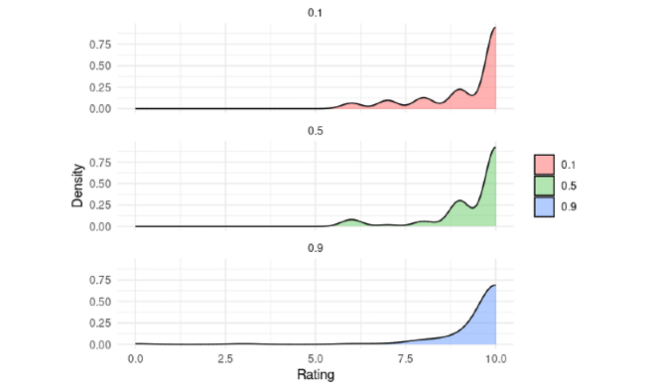
Density plot showing the distribution of tool ratings across different temperature settings (0.1, 0.5, and 0.9).

Post hoc comparisons using Dunn’s test with Holm correction showed no significant difference between temperature levels 0.1 and 0.5 (*P*=0.62) nor between 0.5 and 0.9 (*P*=.14). However, a significant difference was found between 0.1 and 0.9 (*P*=.04), suggesting that higher ratings were associated with the 0.9 temperature condition (Figure 5).

#### Student Satisfaction Versus Gender Pairing

**Figure 6:**
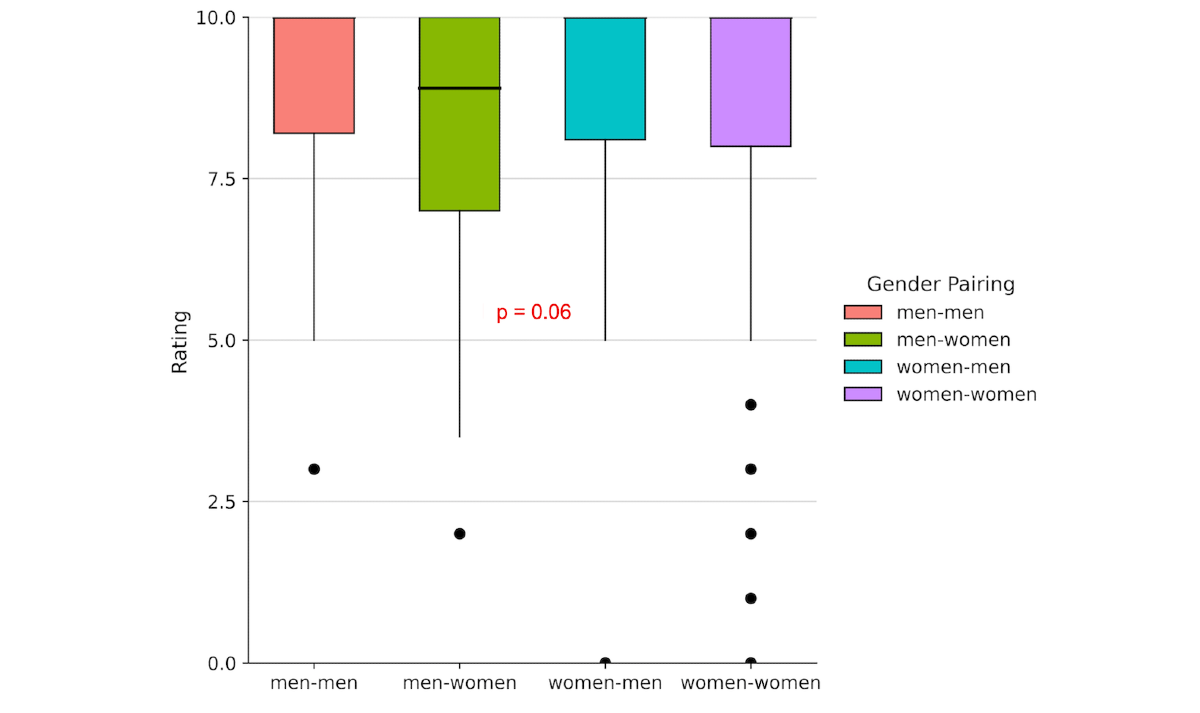
Boxplot showing the distribution of tool ratings by gender pairing between the participant and the virtual simulated patient (VSP).

Regarding the gender pairing between students and VSPs (Figure 6), the Kruskal-Wallis test revealed no statistically significant differences between groups (H=7.41, *P*=.06). Post hoc comparisons using Dunn’s test with Bonferroni correction also showed no significant differences across any of the gender combinations evaluated. These results suggest that neither the participant’s gender nor that of the VSP had a meaningful impact on how the tool was rated.

However, given that the *P* value was close to the conventional threshold for significance, it would be advisable to include a larger sample in future studies to more accurately assess whether gender pairing influences students’ evaluations of the tool.

### Learning Improvement

A total of 100 students completed the optional final questionnaire. Table 4 shows the results obtained the question related to learning improvement.

According to the results obtained, the ability to identify relevant symptoms was mostly agreed (94/100, 94% of students found their ability had increased “a great deal” or “quite a lot”).

**Table 4:** Final questionnaire, item related to learning improvement.

Question: Do you consider that interacting with virtual patients helped you improve your ability to identify relevant symptoms during the clinical interview?	Answers, n (%)
A great deal	43 (43)
Quite a lot	51 (51)
Somewhat	5 (5)
A little	1 (1)
Not at all	0 (0)

**Figure 7:**
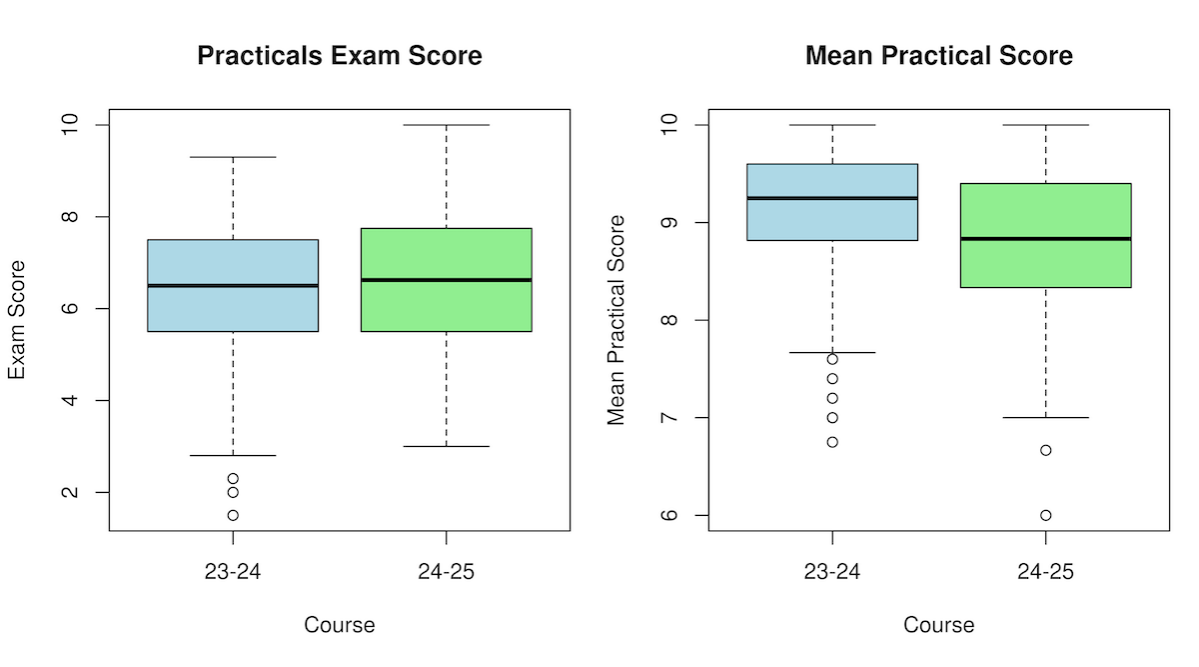
Mark comparison against previous course. Exam: examination.

The analysis of the final practical examination (Figure 7) showed that the mean score obtained in course 2024/2025 (mean 6.67, SD 1.42) was slightly higher than that of course 2023/2024 (mean 6.42, SD 1.56). However, this difference was not statistically significant (W=9297; *P*=.46).

Conversely, the analysis of the average practical session grades revealed that the scores from the 2024/2025 course (VSP-based; mean 8.8, SD 0.77) were significantly lower than those from the 2023/2024 course (paper-based; mean 9.14, SD 0.74; W=12,428; *P*<.001).

### Sentiment Analysis

All interactions with the platform (either student questions or GAI answers) were recorded and further processed using NLP, with the help of the *pysentimiento* library[23]. The output of the library rates the positive, neutral, and negative sentiments of each sentence, normalized so that positiveness + negativeness + neutralness = 1.

**Figure 8:**
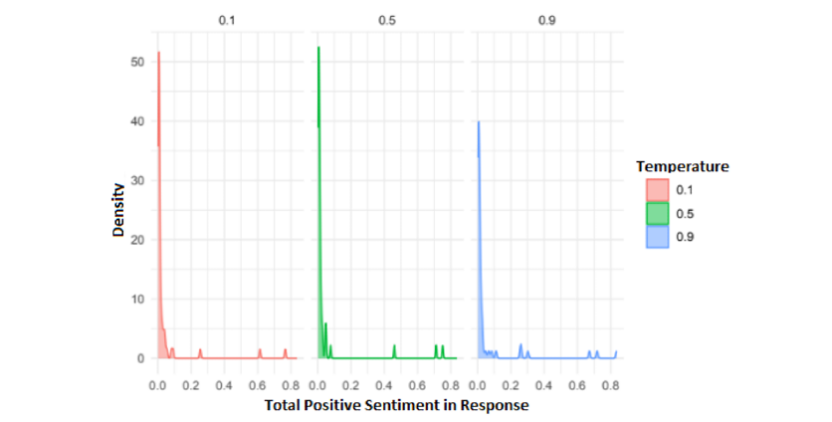
Density plot of total positive sentiment in generative artificial intelligence (GAI) responses, grouped by model temperature (0.1, 0.5, and 0.9).

The first analysis carried out tried to explore whether the emotional tone of the GAI responses was influenced by the temperature parameter of the GAI model. We only show positiveness and negativeness results, since neutralness can be obtained from them. Figure 8 displays the total positive sentiment in responses (median 0.008, IQR 0.003-0.079). The results show a striking concentration of low positive sentiment across all temperature levels, especially at 0.1 and 0.5. Interestingly, temperature 0.9 shows slightly more dispersion, possibly due to more expressive or varied GAI outputs under higher randomness. Despite this, positivity in responses remains generally low, consistent with the structured, clinical nature of the interactions.

**Figure 9:**
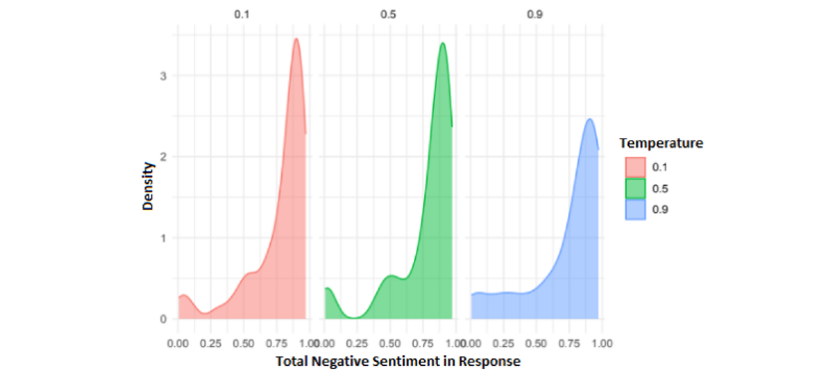
Density plot of total negative sentiment in generative artificial intelligence (GAI) responses, grouped by model temperature (0.1, 0.5, and 0.9).

Figure 9 presents the total negative sentiment in responses, where a clear concentration of high negativity scores was observed across all temperature levels (median 0.890, IQR 0.306-0.961). This was particularly noticeable at temperatures 0.1 and 0.5. These findings may reflect the emotional content inherent in the psychological case scenarios, in which patients often express distressing or symptomatic narratives.

This sentiment configuration shown in Figures 8 and 9 could be partially attributed to the design of the VSPs themselves, as they were intentionally modeled to represent clinical profiles commonly seen in mental health settings. These profiles often contain emotionally charged content, which likely contributes to the predominance of negative sentiment over positive sentiment in the GAI responses. Consequently, higher temperature values may lead the model to deviate from the expected clinical behavior, producing more creative and expressive responses that go beyond the original configuration of the VSPs [28]. This creative drift may result in a more positive tone in the interaction, as the model becomes less constrained by the simulated symptoms or emotional distress typically expected from a psychological patient.

Additional results concerning sentiment analysis are available in Multimedia Appendix 6, together with other statistical results not included in the main document.

### Content Analysis

A total of 1708 valid answers (excluding empty answers, nonalphabetic answers, or answers without meaning) were registered as positive comments (positive aspects found in the tool). The automated content analysis of those comments is detailed in Table 5.

Concerning negative comments (or improvement suggestions), a total of 1604 valid answers were registered (using the same exclusion criteria as for positive comments). Automated content analysis of negative comments is summarized in Table 6.

**Table 5:** Content analysis of positive comments.

Positive aspect	Repetitions, n	Details
Educational usefulness and practical application	~570	Most valued aspect. Users report that the tool helps apply clinical knowledge, practice interviews, and develop professional skills in a safe environment. Universally described as useful, effective, and enriching.
Quality and clarity of the VSP’s^a^ responses	~470	Responses are accurate, clear, and coherent. Relevant for diagnosis, allowing interview progress. Includes completeness, correctness, and clinical utility.
Clarity and fluency in the interaction	~320	Emphasis on conversational naturalness, ease of use, and absence of glitches. Enhances the interview experience and realism.
Engagement, motivation, and dynamic experience	~240	Tool is engaging, maintains interest, and motivates learners. Nonmonotonous interaction supports student engagement and active learning.
Accurate symptom description and diagnostic support	~250	VSP provides rich and detailed symptom descriptions. Aids clinical reasoning and realistic hypothesis formulation.
Perceived improvement and positive comparison	~140	Perceived positive evolution in tool functionality and response quality. Increases satisfaction and perceived quality.
Perceived realism and immersiveness	~120	Interaction closely resembles real interviews. Realism improves pedagogical value and clinical preparation.

^a^VSP: virtual simulated patient.

**Table 6:** Content analysis of negative comments

Suggestion	Repetitions, n	Details
Realism and content of the VSP’s^a^ responses	~110	Suggestions focus on enhancing coherence, depth, and appropriateness of the VSP’s clinical language. Proposals include: avoiding repetition, tailoring responses to age (eg, young children), adding relevant details, and ensuring internal consistency.
Diagnostic clarity and symptom presentation	~82	Many comments highlight difficulties in interpreting symptoms due to the similarity between disorders. Some users report that the patient directly reveals the diagnosis, undermining the clinical exercise. There is a request for more subtle clinical clues and better-differentiated scenarios.
Technical functionality and system errors	~74	Recurrent technical issues are reported: connection failures, GAI^b^ model not being available, automatic deletion of student answers, and the need to reload the activity. In some cases, users are forced to repeat the task.
User interface and navigation	~49	Recommendations include improving navigation, enhancing the visibility of return buttons, enabling users to go back without losing information, and simplifying transitions between patients or tasks.
Linguistic clarity and textual formulation	~37	There is a call to improve the phrasing of both questions and responses. Suggestions include using clearer, more precise language appropriate to students' comprehension level.

^a^VSP: virtual simulated patient.

^b^GAI: generative artificial intelligence.

## Discussion

### Principal Findings

Concerning temperature influence on results, although not all observed effects reached statistical significance, clear trends emerged, particularly when comparing the lowest temperature level tested (0.1) with the highest one (0.9). The results in terms of user satisfaction were significantly higher for the 0.9 setting. This suggests that the temperature parameter may play a meaningful role in shaping students’ perceptions of the interaction.

Contrary to expectations, no significant relationship was found between the number of questions asked during the simulation or the participants’ age and the rating they assigned. However, as one might anticipate, a negative correlation was observed between the number of connectivity failures and the students’ evaluation of the experience.

This suggests that students’ perception of usefulness or satisfaction may not depend on the quantity of interaction, but rather on qualitative aspects, such as the fluidity of the dialogue or the perceived realism of the conversation.

A notable finding of this study is the apparent paradox in academic performance: while GAI-powered VSP implementation (course 2024/2025) led to significantly lower average grades in practical sessions compared to the traditional paper-based method (course 2023/2024), grades obtained in the final practical examination were slightly higher, although not statistically significant. Far from suggesting lower efficacy, we interpret this as evidence that the VSP simulations provide a more demanding and clinically realistic learning challenge. Traditional static paper-based cases reward methodical information retrieval [8], whereas the dynamic VSP tool required students to actively engage in real-time clinical interviewing and hypothesis formulation [6], better mirroring real-world clinical ambiguity [4]. Further randomized experiments are required to draw more reliable conclusions.

### Comparison to Prior Work

Our findings on the influence of the temperature parameter are consistent with those found in previous literature. For instance, the experiments carried out by Davis et al [29] in different clinical research scenarios emphasize the compromise between creativity and consistency of the GAI answers and suggest specific temperature levels depending on the task. Other recent studies warn about the impact of inconsistencies and errors in ChatGPT’s responses on user satisfaction when higher temperature settings are used [30], but in our case, the highest temperature tested (0.9) offered the best results in terms of user satisfaction.

The general evaluation of the VSP platform was highly positive, indicating strong acceptance of this type of simulation in clinical training contexts. In general, this result aligns with previous studies that have highlighted the potential of VSPs to create immersive learning environments that foster the development of clinical reasoning from the early stages of professional training [8].

Compatible with our results, the work presented by Peralta et al [10], based on an experiment with 32 medicine students, found highly valued student perceptions for both realism and consistency of the VSP responses. In particular, the students answered “agree” or “strongly agree” in 91% of the cases for the question “the scenario was realistic and similar to an authentic clinical situation,” and in 94% of the cases for the question “the virtual patient responded appropriately to my actions and questions.”

Focusing on specific aspects, the previous work on VSPs presented by Kamath et al [31] (pharmacology students, n=19) showed strongly positive user satisfaction for most aspects, particularly for “authenticity of patient encounter and consultation” (92.11% of positive responses), but low values for “learning effect of consultation” (47.37% of positive responses). In comparison, our experiments with psychology students agree on high user satisfaction for authenticity (Table 5, row 3: “conversational naturalness,” “realism”) and also offer strongly positive values for learning improvement, with 94% of students answering “a great deal” or “quite a lot” to the question “Do you consider that interacting with virtual patients helped you improve your ability to identify relevant symptoms during the clinical interview?” (Table 4). The difference in this particular result may be related with the specificities of pharmacology and psychology studies.

Another previous study, with medicine students (n=9) is presented by Cross et al [32]. Contrarily to our results, their students found verisimilitude issues and lack of empathy in the VSPs’ answers. Such result may be related to the use of standard values for the temperature parameter (since the experiments were carried out directly from the web interface of ChatGPT) or a too strict definition of the clinical cases.

### Strengths and Limitations

According to the results shown in Table 6, students find the tool helpful, relevant, and motivating. In addition, they particularly valued the realism of the interactions. The most common suggestions, as shown in Table 5, refer to improvements in the clinical language used by the VSPs, increasing the difficulty of the cases, avoiding connection failures, and improving the user interface.

The findings of this study provide preliminary evidence for the feasibility of using LLMs such as GPT-4o to simulate virtual patients in educational settings. The tool was rated positively by most participants, suggesting it can serve as an effective strategy for training fundamental clinical skills—such as conducting psychological interviews or gathering relevant case information—in a safe and controlled environment [6,31].

Moreover, the ability to adjust the model’s temperature setting allows educators to tailor the GAI’s behavior to specific learning objectives, making it possible to design adaptable training experiences that align with the learner’s level of competence and the complexity of the scenario.

Concerning content analysis results, one of the most repeated positive comments was “responses are accurate, clear, and coherent. Relevant for diagnosis, allowing interview progress. Includes completeness, correctness, and clinical utility” (Table 5). On the other hand, the most repeated improvement suggestion was focused on “enhancing coherence, depth, and appropriateness of the virtual patient’s clinical language” (Table 6). Surprisingly, the coherence of the VSPs’ responses was considered both as a strength of the platform and as a topic requiring improvement. That suggests that, according to the students, coherence is a key point in a VSP.

This study has several limitations.

First, this was a cross-sectional, observational study, which limits the ability to draw causal conclusions from the findings. In addition, a potential source of bias was identified in the rating scale: the value “5” appeared as the default option in the evaluation form, making it unclear whether selections of this score were made intentionally or by oversight.

Second, another limitation involves the uneven usage of different VSP profiles and GAI models, which may restrict the generalizability of the results. Future research would benefit from a more balanced distribution of exposure to each virtual character and system configuration.

Third, the study’s design lacked randomization. The comparison of academic performance was quasi-experimental, contrasting the 2024/2025 cohort (which used the VSP tool) against the previous 2023/2024 cohort (which used paper-based cases) rather than using a randomized controlled trial. This nonrandomized approach means we cannot definitively attribute observed differences, or the lack thereof, in academic performance solely to the VSP intervention, as other unmeasured confounding variables between the two academic years may have influenced the findings.

Fourth, sentiment analysis was only focused on 2 topics: first, checking the predominant sentiment in VSP responses (which should be negative to reflect the clinical case situations), and second, determining whether sentiment in student questions influenced sentiment on VSP answers or vice versa (details of results are available in Multimedia Appendix 6). However, deeper analysis is needed to measure how closely the VSP reflects the correct sentiment for each case, following, for example, the guidelines that can be extracted from the study of Cero et al [33].

Finally, special attention should be given to the gender imbalance in the sample, which was composed predominantly of female students. Although no significant differences were found between male and female participants across the main variables, this disparity raises questions about potential gender-related biases in perception or interaction with the system. Future studies should aim to recruit more gender-balanced samples to assess these effects more thoroughly.

### Future Directions

One promising line of inquiry is the integration of multimodal features into virtual patient simulations, including speech recognition, nonverbal communication (ie, gesture recognition), or even animated avatars, to increase realism and bring the experience closer to real clinical encounters. These enhancements would allow researchers and educators to assess not only the verbal content of the interaction but also paraverbal and behavioral cues, which are crucial in clinical practice. Nevertheless, in our experience, the VSPs have mostly been used in classroom settings during in-person practical sessions, where keyboard interaction remains the most reliable and least susceptible to disruption from peer interactions.

Another important direction involves carrying out randomized experiments for direct comparisons between GAI-based training and traditional educational methods, such as working with standardized patients or in-person role-play sessions. This would provide clearer insights into the relative effectiveness of each approach in developing specific clinical competencies, as well as students’ perceived realism, usefulness, and transferability to real-world contexts.

Other future studies may explore the implementation of automated feedback systems or peer-based assessments using the transcripts generated during the interactions. These additions could further enhance the educational potential of GAI-powered simulations in hybrid or fully virtual learning environments.

Finally, this study has shown that the VSP generation tool we have developed offers enough flexibility to be adapted across various specialties within psychology, as well as in medicine and nursing. Currently, the tool is also being used in nursing and pediatrics, and we have received requests to implement it in other fields. Given this positive reception, our future goal is to create a complete hospital metaverse—a shared virtual environment that enables practical training across multiple specialties.

## Appendix

Multimedia Appendix 1 CHERRIES checklist.

Multimedia Appendix 2 First questionnaire.

Multimedia Appendix 3 Second questionnaire.

Multimedia Appendix 4 Ethics review board approval.

Multimedia Appendix 5 Additional details about the VSP platform developed.

Multimedia Appendix 6 Additional statistical results.

## Abbreviations

CHERRIES: Checklist for Reporting Results of Internet E-Surveys
GAI: generative artificial intelligence
LLM: large language model
NLP: natural language processing
UMH: Miguel Hernández University
VSP: virtual simulated patient

## Data Availability

The datasets generated or analyzed during this study are available in the Open Science Framework (OSF) [34]. Documents in this repository are password protected; please contact the corresponding author for more information on how to access the data.
