# Supplementary material for: Using AI-Based Virtual Simulated Patients for Training in Psychopathological Interviewing: Cross-Sectional Observational Study
Source: JMIR Med Educ. 2025 Dec 23;11:e78857. doi: 10.2196/78857 (PMC12775747; doi:10.2196/78857)
Supplement: Multimedia Appendix 2 [file mededu_v11i1e78857_app2.docx]

**QUESTIONNAIRE 1, AFTER EACH VSP SESSION**

THREE QUESTIONS, ONE SCREEN

**SCREEN #1**

**QUESTION #1**

TITLE

What is your level of satisfaction with the interaction with the virtual patient?

*¿Cuál es tu nivel de satisfacción con la interacción con el paciente virtual?*

SUBTITLE

Use a scale from 0 to 10, where 0 means ‘not at all satisfied’ and 10 means ‘completely satisfied’.

*Utiliza una escala del 0 al 10, donde 0 representa "nada satisfecho" y 10 "completamente satisfecho".*

[NUMERIC FIELD, LIMITED TO THE RANGE 0-10]

**QUESTION #2**

TITLE

Reflect on your experience during the interview with the virtual patient.

*Reflexiona sobre tu experiencia en la entrevista con el paciente virtual.*

SUBTITLE

Comment on which aspects of the interaction you found most useful or interesting.

*Comenta qué aspectos de la interacción te resultaron más útiles o interesantes.*

[TEXT FIELD, UNLIMITED SIZE]

**QUESTION #3**

TITLE

Suggestions.

*Sugerencias.*

SUBTITLE

Similarly, comment on which aspects of the interaction you believe could be improved for future sessions

*Del mismo modo, comenta qué aspectos de la interacción crees que se podrían mejorar para futuras sesiones.*

[TEXT FIELD, UNLIMITED SIZE]
