# Supplementary material for: Using AI-Based Virtual Simulated Patients for Training in Psychopathological Interviewing: Cross-Sectional Observational Study
Source: JMIR Med Educ. 2025 Dec 23;11:e78857. doi: 10.2196/78857 (PMC12775747; doi:10.2196/78857)
Supplement: Multimedia Appendix 3 [file mededu_v11i1e78857_app3.docx]

**QUESTIONNAIRE 2, AFTER THE LAST SESSION**

14 QUESTIONS, FIVE SCREENS

**SCREEN #1**

Improvement in active learning: identification and differential diagnosis

*(Mejora en el aprendizaje activo: identificación y diagnóstico diferencial)*

**QUESTION #1.1 (ONLY QUESTION USED FOR THE CURRENT STUDY**

TITLE

Do you consider that interacting with the virtual patients (VPs) helped you improve your ability to identify relevant symptoms during the clinical interview?

*¿Consideras que la interacción con los pacientes virtuales (PV) te ayudó a mejorar tu capacidad para identificar síntomas relevantes durante la entrevista clínica?*

[OPTIONS: [“A lot”, “Quite a lot”, “Somewhat”, “A Little”, “Not at all”]

*(“Mucho”, “Bastante”, “Algo”, “Poco”, “Nada”)*

**QUESTION #1.2**

TITLE

To what extent did the simulation with the virtual patient (VP) enable you to differentiate between possible diagnoses?

*(¿En qué medida la simulación con el PV te permitió diferenciar entre distintos diagnósticos posibles?)*

[OPTIONS: [“A lot”, “Quite a lot”, “Somewhat”, “A Little”, “Not at all”]

*(“Mucho”, “Bastante”, “Algo”, “Poco”, “Nada”)*

**QUESTION #1.3**

TITLE

Do you think the VP’s responses reflected realistic behavior that facilitated your clinical reasoning?

*(¿Crees que las respuestas del PV reflejaban un comportamiento realista que facilitó tu razonamiento clínico?)*

[OPTIONS: “Strongly agree”, “Agree”, “Neutral”, “Disagree”, “Strongly disagree”]

*(“Totalmente de acuerdo”, “De acuerdo”, “Neutral”, “En desacuerdo”, “Totalmente en desacuerdo”)*

**SCREEN #2**

Satisfaction and motivation with the tool

*Satisfacción y motivación con la herramienta*

**QUESTION #2.1**

TITLE

How would you rate your overall level of satisfaction with the experience of using virtual patients (VPs) during the practicum?

*(¿Cómo calificarías tu nivel de satisfacción general con la experiencia de utilizar PV durante las prácticas?)*

[Likert, from 1 (Very dissatisfied) to 5 (Very satisfied)]

*[Likert, desde 1 (Muy insatisfecho) hasta 5 (Muy satisfecho)]*

**QUESTION #2.2**

TITLE

Did you feel more motivated to participate in the practicum when interacting with VPs compared to written cases?

(*¿Te sentiste más motivado/a para participar en las prácticas al interactuar con PV que con casos escritos?)*

[OPTIONS: [“Yes, a lot”, “Yes, somewhat”, “Neutral”, “A Little”, “Not at all”]

*(“Sí, mucho”, “Sí, algo”, “Neutral”, “Poco”, “Nada”)*

**SCREEN #3**

Evaluation of the use of artificial intelligence (AI) in the clinical context

*Valoración del uso de inteligencia artificial (IA) en el contexto clínico*

**QUESTION #3.1**

TITLE

Do you think that AI-based virtual patients (VPs) created with ChatGPT can complement or improve simulations with standardized patients (actors)?

*(¿Crees que los PV creados con ChatGPT pueden complementar o mejorar las simulaciones con pacientes estandarizados (actores)?)*

[OPTIONS: [“Yes, completely”, “Yes, partially”, “Neutral”, “Not much”, “Not at all”]

*(“Sí, completamente”, “Sí, algo”, “Neutral”, “No mucho”, “No en absoluto”)*

**QUESTION #3.2**

TITLE

Would you recommend the use of AI-based VPs for other clinical courses?

(*¿Recomendarías el uso de PV basados en IA para otras asignaturas clínicas?)*

[OPTIONS: [“Yes”, “No”]

*(“Sí”, “No”)*

**SCREEN #4**

Participation and use during the sessions

*Participación y uso durante las sesiones*

**QUESTION #4.1**

TITLE

How often did you use the virtual patient to practice your clinical skills during the sessions?

*(¿Con qué frecuencia utilizaste al paciente virtual para practicar tus habilidades clínicas durante las sesiones?*

[OPTIONS: [“In every session”, “In most sessions”, “In some sessions”, “In few sessions”, “Never”]

*(“En todas las sesiones”, “En la mayoría de las sesiones”, “En algunas sesiones”, “En pocas sesiones”, “Nunca”)*

**QUESTION #4.2**

TITLE

On average, how much time did you spend interacting with the virtual patient in each session?

*(¿Cuánto tiempo, en promedio, dedicabas a interactuar con el paciente virtual en cada sesión?)*

[OPTIONS: [“More than 30 minutes”, “Between 15 and 30 minutes”, “Less than 15 minutes”]

*(“Más de 30 minutos”, “Entre 15 y 30 minutos”, “Menos de 15 minutos”)*

**QUESTION #4.3**

TITLE

How many times did you consult the virtual patient in a clinical case before formulating a diagnosis?

*(¿Cuántas veces consultaste al paciente virtual en un caso clínico antes de formular un diagnóstico?)*

[OPTIONS: [“1-2 times”, “3-4 times”, “More than 4 times”]

*(“1-2 veces”, “3-4 veces”, “Más de 4 veces”)*

**QUESTION #4.4**

TITLE

On average, how many questions did you ask the virtual patient for each clinical case?

*(¿Cuántas preguntas le realizaste al paciente virtual por cada caso clínico en promedio?)*

[OPTIONS: [“Less than 5 questions”, “Between 5 and 10 questions”, “More than 10 questions”]

*(“Menos de 5”, “Entre 5 y 10”, “Más de 10”)*

**SCREEN #5**

Participation and use during the sessions

*Participación y uso durante las sesiones*

**QUESTION #5.1**

TITLE

Did you find it intuitive and easy to interact with the virtual patient during the practical activities?

*(¿Te resultó intuitivo y sencillo interactuar con el paciente virtual durante las actividades prácticas?)*

[OPTIONS: “Strongly agree”, “Agree”, “Neutral”, “Disagree”, “Strongly disagree”]

*(“Totalmente de acuerdo”, “De acuerdo”, “Neutral”, “En desacuerdo”, “Totalmente en desacuerdo”)*

**QUESTION #5.2**

TITLE

"Were the virtual patient’s responses useful for identifying errors and improving your clinical reasoning?"

*(¿Las respuestas del paciente virtual fueron útiles para identificar errores y mejorar tu razonamiento clínico?)*

[OPTIONS: [“Yes, a lot”, “Yes, somewhat”, “Neutral”, “A Little”, “Not at all”]

*(“Sí, mucho”, “Sí, algo”, “Neutral”, “Poco”, “Nada”)*

**QUESTION #5.3**

TITLE

"How would you rate your interest in VP-based practical activities compared with traditional methods (written paper-based cases)?"

*(¿Cómo calificarías tu interés en las prácticas con PV respecto a las actividades con métodos tradicionales (casos descritos en papel?)*

[OPTIONS: [“Much higher”, “Somewhat higher”, “The same”, “Somewhat lower”, “Much lower”]

*(“Mucho mayor”, “Algo mayor”, “Igual”, “Algo menor”, “Mucho menor”)*
