# Supplementary material for: Using AI-Based Virtual Simulated Patients for Training in Psychopathological Interviewing: Cross-Sectional Observational Study
Source: JMIR Med Educ. 2025 Dec 23;11:e78857. doi: 10.2196/78857 (PMC12775747; doi:10.2196/78857)
Supplement: Multimedia Appendix 4 [file mededu_v11i1e78857_app4.pdf]

Dr. D. César Fernández Peris
Dpto. Psicología de la Salud

Elche, 7 de octubre de 2025

Investigador/a Principal	César Fernández Peris	
Tipo de actividad	Proyecto Nacional	
Título del proyecto	Pacientes virtuales basados en Inteligencia Artificial y con características específicas de género para la formación en medicina y enfermería sobre entornos gamificados.	
Alcance de la evaluación	☒ Proyecto completo ☐ Evaluación parcial (subproyecto/provisional) ☐ Adenda un de proyecto previamente autorizado	
Códigos GIS estancias donde se desarrolla la actividad	-	
**Evaluación de riesgos laborales**	**Conforme (Autodeclaración)**	
**Evaluación DNSH**	**No procede**	
**Evaluación ética uso muestras biológicas humanas**	**No solicitado**	
**Evaluación ética humanos**	**Favorable**	
**Evaluación ética animales**	**No solicitado**	
Número de expediente	2025/178269	
Código provisional	250116113801	
**Código de autorización COIR**	**DPS.CFP.250116**	
**Caducidad**	**5 años**	

Se considera que el presente proyecto carece de riesgos laborales significativos para las personas que participan en el mismo, ya sean de la UMH o de otras organizaciones.

No se ha evaluado el uso de muestras biológicas humanas porque no se ha solicitado, ni se ha considerado necesario en base a la información aportada.

No se ha evaluado el uso de animales en un proyecto de investigación porque no se ha solicitado, ni se ha considerado necesario en base a la información aportada.

La evaluación de la participación de voluntarios humanos en un proyecto de investigación, desde el punto de vista ético, es favorable.

Por todo lo anterior, el dictamen del CEII es **favorable.**

Atentamente,

Fdo. digitalmente por:
Alberto Pastor Campos
Secretario CEII
Vicerrectorado Investigación y Transferencia

Javier Sáez Valero
Presidente CEII
Vicerrectorado Investigación y Transferencia

Código Seguro de Verificación(CSV): UMHaQORsP5MUcSdtPCpdSSGc
Copia auténtica de documento firmado electrónicamente. Puede verificar su integridad en https://sede.umh.es/csv
Firmado por ALBERTO PASTOR CAMPOS el día 2025-10-08
Firmado por JAVIER SAEZ VALERO el día 2025-10-08

Información adicional:

- En caso de que la presente actividad esté financiada y se gestione a través del servicio SGI-OTRI de la UMH, le recordamos que, para poder llevar a cabo dicha actividad en las instalaciones de la UMH, además del dictamen de la OEP, es necesario contar con la autorización del representante institucional. Esta gestión se realiza a través de SGI-OTRI, quien gestiona las correspondientes prestaciones de servicio, contratos /convenios y proyectos de investigación.
- En caso de que la presente actividad se desarrolle total o parcialmente en otras instituciones es responsabilidad del investigador principal solicitar cuantas autorizaciones sean pertinentes, de manera que se garantice, al menos, que los responsables de las mismas están informados.
- Le recordamos que durante la realización del proyecto debe cumplir con las exigencias en materia de prevención de riesgos laborales. En concreto: las recogidas en el plan de prevención de la UMH y en las planificaciones preventivas de las unidades en las que se integra la investigación. Igualmente, debe promover la realización de reconocimientos médicos periódicos entre su personal; cumplir con los procedimientos sobre coordinación de actividades empresariales en el caso de que trabaje en el centro de trabajo de otra empresa o que personal de otra empresa se desplace a las instalaciones de la UMH; y atender a las obligaciones formativas del personal en materia de prevención de riesgos laborales. Le indicamos que tiene a su disposición al Servicio de Prevención de la UMH para asesorarle en esta materia.

La información descriptiva básica de la presente actividad de investigación será incorporada al repositorio público de proyectos autorizados por la Oficina de Investigación Responsable de la Universidad Miguel Hernández. También se puede acceder a través de https://oir.umh.es/solicitud-de-evaluacion/proyectos-de-investigacion/

Código Seguro de Verificación(CSV): UMHaQORsP5MUcSdtPCpdSSGc
Copia auténtica de documento firmado electrónicamente. Puede verificar su integridad en https://sede.umh.es/csv
Firmado por ALBERTO PASTOR CAMPOS el día 2025-10-08
Firmado por JAVIER SAEZ VALERO el día 2025-10-08
