# Supplementary material for: Using AI-Based Virtual Simulated Patients for Training in Psychopathological Interviewing: Cross-Sectional Observational Study
Source: JMIR Med Educ. 2025 Dec 23;11:e78857. doi: 10.2196/78857 (PMC12775747; doi:10.2196/78857)
Supplement: Multimedia Appendix 5 [file mededu_v11i1e78857_app5.docx]

**Using AI-based virtual simulated patients for training in psychopathological interviewing: cross-sectional observational study.**

**DETAILS ABOUT THE VSP PLATFORM DEVELOPED**

Figure 1 details the software architecture of the VSP interview module, demonstrating the integration and coordination of two GAI models. In brief, whenever a student poses a question to the VSP, the question (along with the full history of prior interactions and contextual patient information) is sent to the primary GAI model. If the model returns an error (e.g., due to internet connectivity issues or server overload), the student is informed and prompted to repeat the question. If the model responds correctly, its answer is forwarded to the supervision GAI (also with the full interaction history and patient context). This second model evaluates whether the initial response is appropriate, based on an adjustable confidence threshold. If the supervision model validates the response, it is presented to the student as the VSP’s reply. If not, a new response is requested from the primary GAI model, with a reduced temperature parameter to increase the likelihood of a more focused and contextually accurate reply, minimizing hallucinations.

**VIRTUAL PATIENT**

Hello doctor

**…**

**…**

EXTRA INFO

END INTERVIEW & QUIZ

**Open AI / Mistral**

Main API call

**Mistral / Open AI**

Supervising API call

question

+ context

+ history

ANSWER

ERROR

*notify user and ask to repeat question*

PASS

FAIL

*reduce temperature and repeat API call*

*show result*


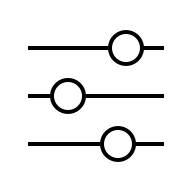


adjustable threshold

Figure 1: Software architecture

Regarding the GAI models used, OpenAI (ChatGPT) was used as the primary model, and Mistral was used for supervision, although the platform allows for easy interchangeability. Access to both models was provided via API calls. The specific models used were gpt-4o-2024-08-06 and gpt-4o-mini-2024-07-18 from OpenAI, and mistral-large-latest from Mistral.

Concerning the tool developed for docents or instructors to create and edit VSPs, Figure 2 illustrates the information required to define a VSP. Specifically, instructors are asked to provide: 1) basic patient information, such as name, photo, age, and gender; 2) detailed information about the clinical case, including symptoms, their duration, and both personal and family medical history; 3) patient behavior (to enhance the realism of the interaction, it is possible to define the type of language the patient should use, the length of responses, and the conversational tone); 4) optionally, supplementary documentation such as manuals or other relevant information to the students; 5) the questions students must answer at the end of the interview, which may include multiple-choice questions (with immediate feedback), free-text responses, or other formats.

Using this information, a fully functional VSP is generated. The platform also enables an easy testing and refinement process to allow instructors to improve their VSP cases.

**PATIENT INFO**

Photo/image.

Name.

Age.

Gender…

**CLINICAL CASE**

Fully detailed!

Symptoms.

Previous diseases.

Family history…

**PATIENT BEHAVIOR**

Answer style.

Answer length.

Language used…

**EXTRA INFO**

Manuals.

Clinical tests.

Previous history…

**STUDENT QUIZ**

Multiple choice.

Open text.

Other…

VSP generator

**VIRTUAL PATIENT**

How do you feel?

**…**

**…**

EXTRA INFO

END INTERVIEW & QUIZ

Figure 2: VSP generator: inputs required.
