# Supplementary material for: Using AI-Based Virtual Simulated Patients for Training in Psychopathological Interviewing: Cross-Sectional Observational Study
Source: JMIR Med Educ. 2025 Dec 23;11:e78857. doi: 10.2196/78857 (PMC12775747; doi:10.2196/78857)
Supplement: Multimedia Appendix 6 [file mededu_v11i1e78857_app6.docx]

**Using AI-based virtual simulated patients for training in psychopathological interviewing: cross-sectional observational study.**

**ADDITIONAL STATISTICAL RESULTS**

**Interview length**

Interview length was analyzed for relationships with student gender and with GAI model temperature settings.

Figure 1 illustrates the distribution of the number of questions posed by participants (interview length), segmented by gender. Both groups showed a peak around nine questions, which also represented the median value. However, the distributions differed slightly: female participants tended to cluster more tightly between 8 and 12 questions, with a higher mean (mean = 10.2, 95CI [9.97–10.43])), while male participants showed greater variability and a slightly lower mean (mean = 9.5, 95CI [9.01–9.99]), although both groups were centered around similar values.


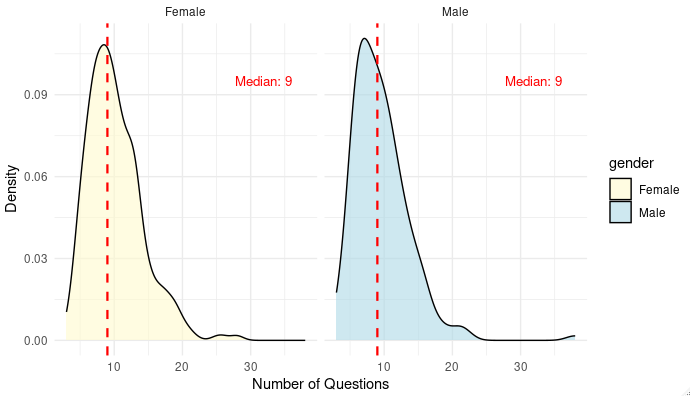


Figure 1. Distribution of the number of questions asked during the interactions by participant gender. The dashed red line indicates the median for each group.

The distribution of the number of questions asked by participants was also analyzed across the different temperature settings of the model.

As Figure 2 shows, at a temperature of 0.1, the number of questions tended to be more concentrated, with a higher density between 5 and 10 questions. At 0.5, the distribution widened slightly, peaking around 10 questions. Finally, at a temperature of 0.9, greater variability was observed, with participants tending to ask slightly more questions overall, though the responses were more widely dispersed.


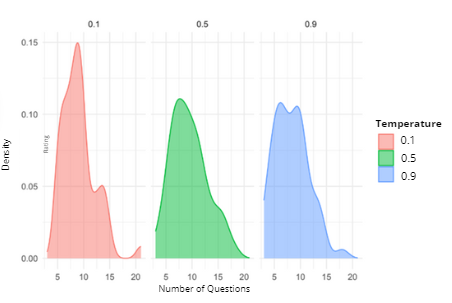


Figure 2. Density plot showing the distribution of the number of questions asked by participants at different GAI temperature settings (0.1, 0.5, and 0.9).

**Sentiment analysis (additional results)**

Although the students’ questions should not be influenced by the GAI *temperature* parameter, we also carried out a sentiment analysis on these questions. Figure 3 shows the distribution of total positive sentiment in questions (Median = 0.042, IQR = 0.018–0.099). The scores are generally low across all temperature levels, though a slightly wider distribution is observed at higher temperatures, suggesting that increased randomness from the IA may encourage a broader range of emotionally positive phrasing from students.


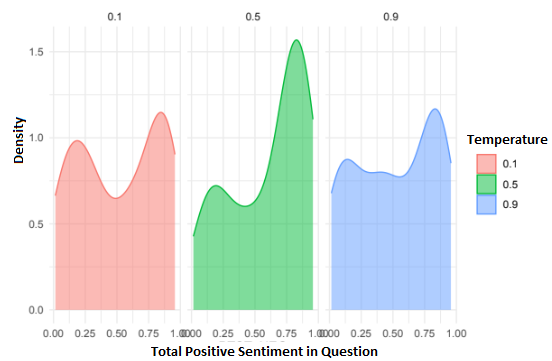


#### Figure 3. Density plot of total positive sentiment in student questions, grouped by model temperature.

Such (theoretically indirect) effect of the *temperature* parameter in the sentiment of students’ questions was further studied by measuring the influence of the sentiment of students’ questions on the sentiment of GAI’s answers and vice versa. In other words, was the mood or the student affected by the mood of the IA, or the other way round?

Table 1 summarizes the sentiments found both in students’ questions (Q) and GAI’s responses (R), showing the median value, as well as Q1 and Q3 quartiles for positiveness, negativeness and neutralness. Please note that these values are computed per each single sentence (student question or GAI response) and then averaged, as opposed to the values shown on the previous analyses, where the sentiment analysis was computed on the aggregated text of all questions and the aggregated text of all responses of each student-GAI conversation.

| **Sentiment** | **Median** | **Q1** | **Q3** |
| --- | --- | --- | --- |
| Positive (Q) | 0.0419 | 0.0184 | 0.0987 |
| Positive (R) | 0.0076 | 0.0033 | 0.0794 |
| Negative (Q) | 0.2860 | 0.1017 | 0.6372 |
| Negative (R) | 0.8903 | 0.3055 | 0.9610 |
| Neutral (Q) | 0.5667 | 0.3145 | 0.7383 |
| Neutral (R) | 0.0913 | 0.0353 | 0.3309 |

Table 1. Summary statistics of sentiment values in questions and responses

To determine the influence of the student sentiment on the GAI sentiment, we examined the correlation between the positive, negative, and neutral sentiment proportions of each student question (Q_n_) and its corresponding GAI response (R_n_).

As shown in Figure 4, the correlations were moderate for positive sentiment (r = 0.40 [0.37, 0.41], p < .001) and lower for negative sentiment (r = 0.25 [0.23, 0.27], p < .001), with only a weak correlation for neutral sentiment (r = 0.11 [0.10, 0.14], p < .001). These patterns are better understood in light of the distribution of sentiment values. Most interactions displayed very low levels of positive sentiment, especially in GAI responses, where the median was close to zero. This imbalance likely constrains the strength of alignment between question and response tone. Meanwhile, negative sentiment was more prevalent, especially in responses (Median = 0.890, IQR = 0.306–0.961), suggesting a more emotionally intense style of reply from the model, even when user input was moderately negative. Neutral sentiment dominated user input but was less frequent in the GAI responses, indicating a general tendency of the model toward more affect-laden replies.

These results may be partially influenced by the way the VSPs were configured. Given that the simulated cases represented psychological patients, their conversational tone was designed to reflect emotional vulnerability, discomfort, or distress. This design choice likely contributed to the prevalence of negative sentiment in their responses, as many of their narratives involved fear, anxiety, or sadness.

Such emotional tone aligns with the clinical realism expected in training scenarios but may also have skewed the sentiment distribution, leading to fewer positively valenced or neutral responses from the model, even when students' questions were neutral or supportive in nature.


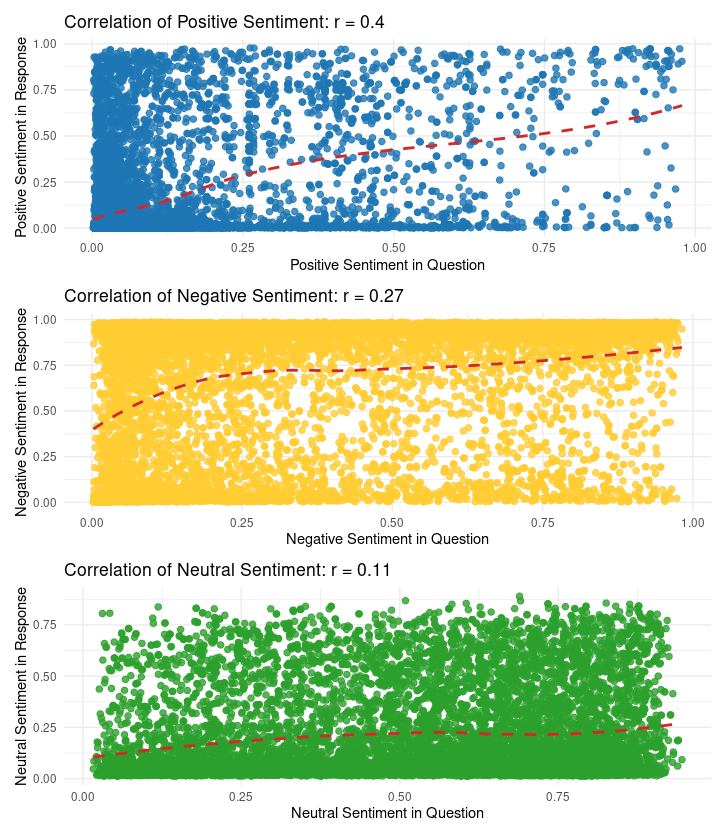


#### Figure 4. Correlation plots for sentiment in student question Q_n_ and sentiment in GAI’s response R_n_ (influence of student mood on GAI mood).

We carried out the opposite analysis (effect of GAI response R_n_ on the next student question Q_n+1_) to study the effect of the GAI mood on the student mood. While this approach does not provide definitive evidence of causality, it offers insight into the extent to which the tone of the GAI may influence the user's emotional framing in the following turn.

Pearson correlations were calculated between each sentiment dimension of the response and the subsequent question. The results shown in figure 5 indicated a moderate positive correlation for positive sentiment (r = 0.18 [0.16, 0.20], p < .001), a weaker but significant correlation for negative sentiment (r = 0.11 [0.9, 0.13], p < .001), and a very small correlation for neutral sentiment (r = 0.05 [0.03, 0.07, p < .001). These findings suggest that, to some extent, the emotional tone of the GAI's reply may shape the sentiment of the next question posed by the student, especially for positive expressions.


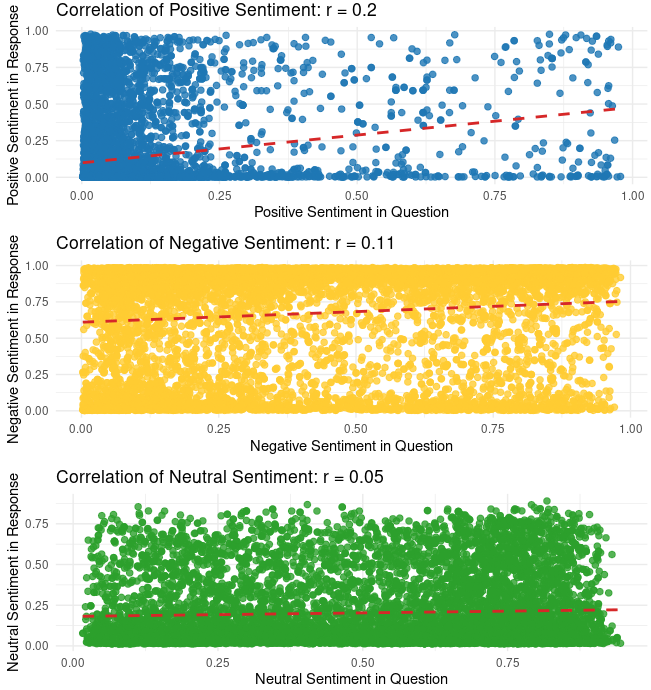


#### Figure 5. Correlation between the sentiment of the GAI response and the sentiment of the student’s following question.

These findings suggest that although the dominant direction of influence flows from student to GAI, there is also a subtle feedback effect in which the model’s tone may help guide the emotional tone of the ongoing interaction—particularly in terms of fostering positive affect.
